# Supplementary material for: The interactome of intact mitochondria by cross-linking mass spectrometry provides evidence for coexisting respiratory supercomplexes
Source: Mol Cell Proteomics. 2017 Dec 8;17(2):216–32. doi: 10.1074/mcp.RA117.000470 (PMC5795388; doi:10.1074/mcp.RA117.000470)
Supplement: Supplemental Data [file supp_RA117.000470_133922_0_supp_23978_fzffwf.zip › spectra_annotation/mito_DR_spectra_annotation/47-1-8-1-5-1.pdf]

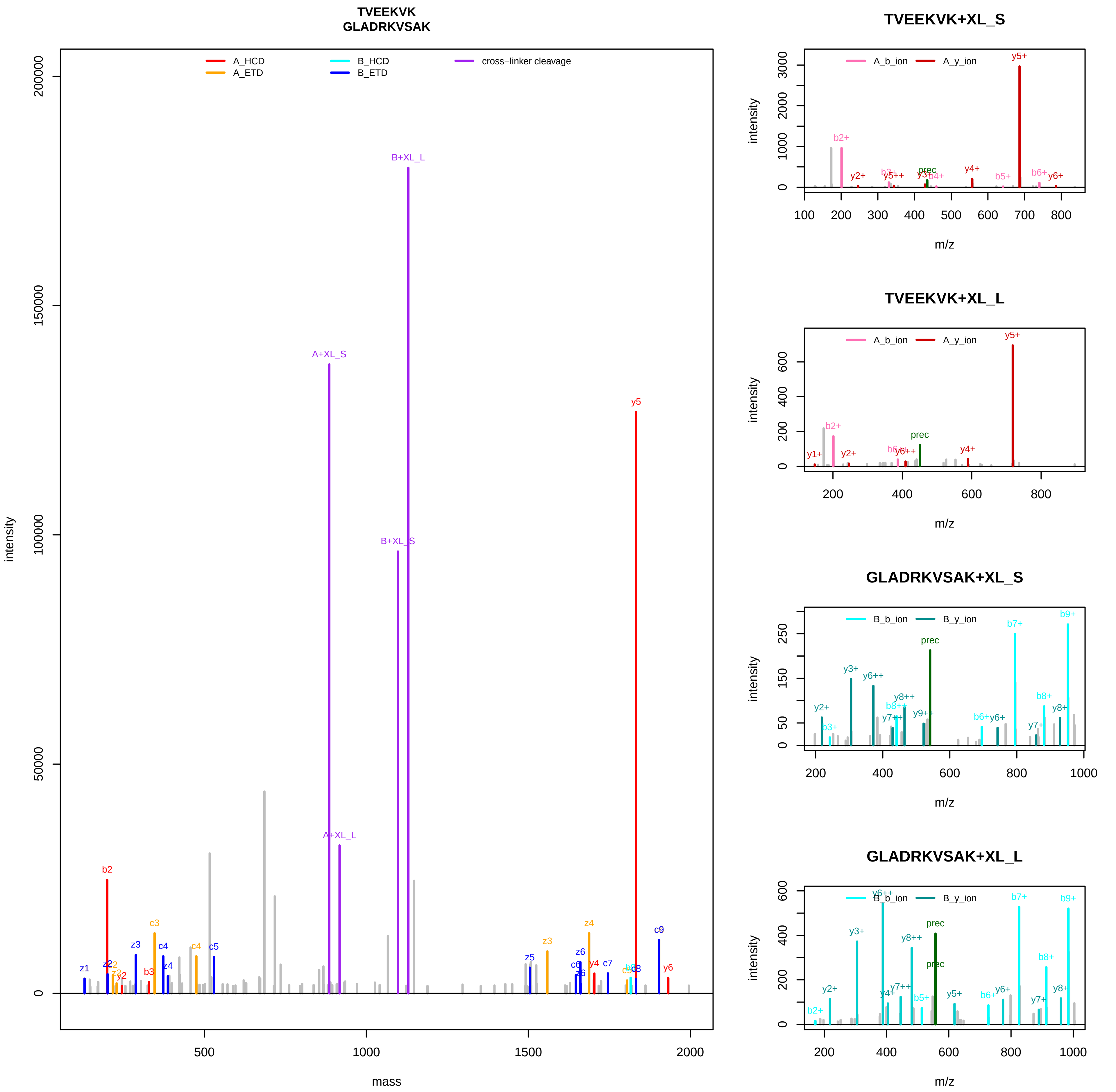
TVEEKVK
GLADRKVSAK
A_HCD
A_ETD
B_HCD
B_ETD
cross−linker cleavage
B+XL_L
A+XL_S
B+XL_S
A+XL_L
y5
b2
c3
c4
c5
z1
z2
z3
z4
b3
y2
z3
z4
z5
z6
c6
c7
c8
c9
y4
y6
intensity
mass
0
50000
100000
150000
200000
500
1000
1500
2000
TVEEKVK+XL_S
TVEEKVK+XL_L
GLADRKVSAK+XL_S
GLADRKVSAK+XL_L
A_b_ion
A_y_ion
B_b_ion
B_y_ion
prec
m/z
y5+
y4+
b2+
y2+
b6+
y6+
b5+
y1+
y6++
b7+
b9+
b8+
y8+
y3+
y6++
y8++
b8++
y7++
y9++
b6+
y6+
y7+
b3+
y2+
b2+
y4+
b5+
y5+
